# Acute Bilateral Swelling of the Parotid Gland After General Anesthesia in Lateral Decubitus Position

**DOI:** 10.7759/cureus.29154

**Published:** 2022-09-14

**Authors:** Kiranmai Chidipothu, Neeta Chaudary Verma, Sheetal Madavi, Jui A Jadhav

**Affiliations:** 1 Anaesthesiology, Jawaharlal Nehru Medical College, Datta Meghe Institute of Medical Sciences, Wardha, IND

**Keywords:** anesthesia, anesthesia mumps, lateral decubitus position, sialadenitis, salivary gland swelling

## Abstract

A rare but well-known anesthetic side effect is acute parotid gland enlargement after general anesthesia, sometimes known as anesthesia mumps or acute post-operative sialadenitis. Acute dehydration, obstruction of glandular excretory ducts caused by the position of the patient, and/or medications such as atropine that increase saliva viscosity have all been proposed as potential causes, while the specific cause is still unknown. We present a case of a 41-year-old patient who underwent a right open anatrophic pyelolithotomy for a staghorn calculus in the left lateral decubitus position and had swelling in the right and left preauricular and postauricular regions, which had progressed to the angle of the mandible post-operatively.

## Introduction

During anesthesia, acute temporary enlargement of one or more salivary glands is unusual. In the literature, it is referred to as "anesthesia mumps" when the parotid gland is affected. It may be unilateral or bilateral and usually painless and resolves on its own within a few hours or days without any lasting effects [1-3]. Here, we discuss a case of bilateral parotid gland swelling following right open anatrophic pyelolithotomy for staghorn calculus in the left lateral decubitus position in a middle-aged male.

## Case presentation

A 41-year-old male weighing around 85 kg was posted for right open anatrophic pyelolithotomy for a staghorn calculus under general anesthesia. According to the American Society of Anesthesiologists (ASA), his physical status was class II, non-smoker, and social alcoholic drinker. His medical history was insignificant; he had no known allergic history. A normal dentition with three-finger mouth opening and Mallampati grade II, as well as a complete range of neck and temporomandibular joint movement, were revealed during the preoperative examination of airway assessment. The cardiovascular and respiratory systems were normal. Laboratory tests were within normal limits. The electrocardiogram (ECG) showed a normal sinus rhythm and a clear chest X-ray. A wide-bore intravenous (IV) access was obtained in the operation theater (OT), and standard anesthetic monitoring devices, including an electrocardiogram, a saturation probe, and a noninvasive blood pressure monitoring, were attached.

Before the induction of anesthesia, injection of glycopyrrolate 0.2 mg IV, injection of midazolam 2 mg IV, and injection of butorphanol 1 mg were administered as premedication. The patient was induced with injection of propofol 2 mg per kg and injection of vecuronium 0.1 mg per kg and intubated with number 8 endotracheal tube after three minutes of mask ventilation and was then placed on mechanical ventilation volume control mode with 8 mL per kg tidal volume, 14 breaths per minute respiratory rate, and 5 cm positive end expiratory pressure (PEEP) of H2O. The patient was positioned in the left lateral decubitus posture 10 minutes after endotracheal insertion. A head ring and appropriate padding were used to support the patient's head.

**Figure 1:**
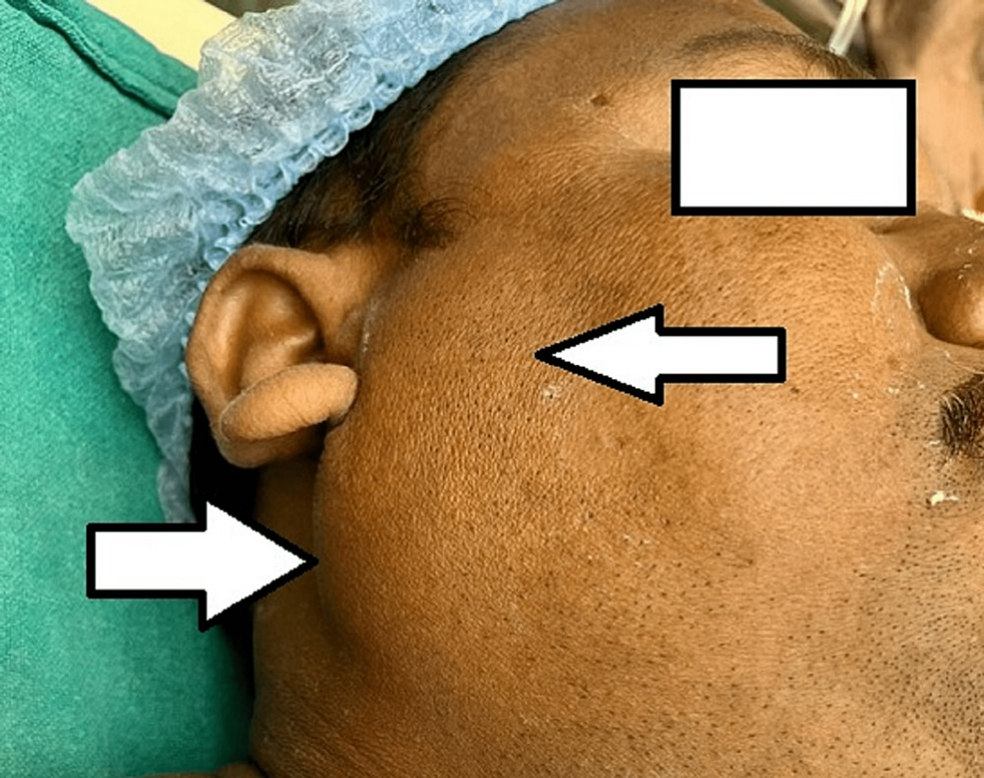
Right parotid gland swelling immediate post surgery.

**Figure 2:**
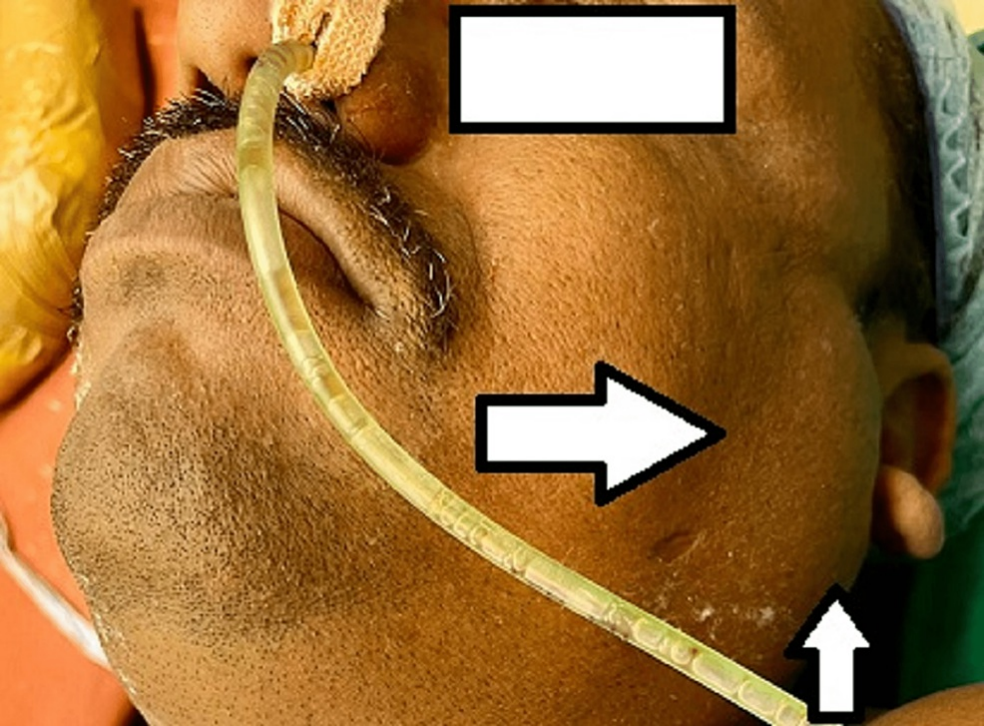
Left parotid gland swelling immediate post surgery.

While the muscle relaxant was intermittently supplied, the anesthesia was maintained using oxygen flow at the rate of 2 L per minute, nitrous oxide flow at the rate of 2 L per minute, and sevoflurane at 0.8% concentration with minimum alveolar concentration (MAC) of 1.0. Throughout the surgery, fluid replacement was maintained by crystalloids and colloids; urine output was measured at a rate of 0.5-1 mL per hour, and 400 mL of blood loss was noted overall during the four-hour-long procedure. After the procedure, the patient was placed back in the supine position, reversed with injection myopyrolate, and extubated. The entire procedure was uneventful. At the end of the surgery, preauricular and postauricular swelling was seen on both sides, reaching the angle of the mandible (Figure 1 and Figure 2).

No crepitations were felt and no reddish lesions or no local rise of temperature over the enlarged area. The IV administration of hydrocortisone 100 mg and dexamethasone injection 8 mg was done. The patient was transferred to post-operative care unit (PACU) for observation. Both parotid glands were hyperechogenic and had a modest parotid duct dilatation, according to portable ultrasonography performed in the PACU. The patient didn't express any discomfort after recovering. The swelling subsided within 24-48 hours.

## Discussion

Bilateral or unilateral parotid gland swelling can be noticed during surgery or in early post-operative period. There are no long-term repercussions from this swelling, which will go down on its own over the course of a few hours or days [1-4]. Although several theories have been put out, the etiology has not yet been established. Systemic dehydration, retrograde airflow through Stensen's duct orifice during coughing and straining while under anesthesia, pooling of secretions that obstruct the salivary ducts, and preoperative use of different medications such as atropine, morphine, and succinylcholine are frequently described causes [3,5-8].

In a case following hip replacement surgery under regional anesthesia, Pirat et al. observed bilateral submandibular salivary gland enlargement, and they suggested the following two explanations: caused by the use of perioperative vasopressors, hypovolemia, or sympathetic stimulation [4]. Another explanation is ischemic sialadenitis caused by glandular ischemia. The compression of vasculature either arterial or venous may impair blood flow to the gland, which can result in ischemia and ischemic sialadenitis. Ischemia-related sialadenitis typically presents as a painful unilateral swelling; red hemorrhagic patches over that area are frequently associated with the clinical appearance [3,9].

The head placement during long surgical procedures may be one of the causes. Several cases of enlarged salivary glands have been notified, particularly after protracted procedures carried out while seating or lying flat [1,2,10]. After extensive procedures in the lateral decubitus position, compressive pressure blocking Stensen's duct may cause obstructive acute transitory sialadenopathy.

A 35-year-old patient experienced swelling in the left parotid gland after being in the left lateral decubitus posture during a laparoscopic nephrectomy, according to Postaci et al. [11]. Stasis in Stensen's duct due to dehydration was not considered to be a potential cause of swelling in our case because the patient received enough intraoperative hydration. When the enlarged area was palpated, no signs of inflammation, infection, or crepitations were noticed, symptoms that are frequently connected to subcutaneous emphysema. No medications known to cause salivary gland swelling were given at any point during the anesthesia.

In our patient, induction was uneventful, and there were no known disorders in our case. The swelling was not deemed to be hemorrhagic or indicative of ischemic sialadenitis because it was painless. In this case, because of the patient's position, we thought that acute obstructive transitory parotitis associated with direct compression is the causality. Post-operatively, the swelling began to subside, and 24-48 hours later, it was completely resolved.

## Conclusions

Lastly, surgeries performed in the lateral decubitus position may result in acute salivary gland swelling. In order to avoid this during lengthy operations, we advise utilizing adaptive-shaped soft pads to adjust the head and neck posture. By doing this, mechanical compression of the parotid gland and its duct can be prevented. The anesthesiologist, surgeon, and patient must all comprehend that in this particular clinical situation, the edema is of minimal clinical significance and will go away on its own with appropriate symptomatic care.
